# Genetic diversity, breed composition and admixture of Kenyan domestic pigs

**DOI:** 10.1371/journal.pone.0190080

**Published:** 2018-01-22

**Authors:** Fidalis Denis Mujibi, Edward Okoth, Evans K. Cheruiyot, Cynthia Onzere, Richard P. Bishop, Eric M. Fèvre, Lian Thomas, Charles Masembe, Graham Plastow, Max Rothschild

**Affiliations:** 1 Nelson Mandela Africa Institution of Science and Technology, Arusha, Tanzania; 2 USOMI Limited, Hardy Post, Karen, Nairobi, Kenya; 3 International Livestock Research Institute, Nairobi, Kenya; 4 Department of Veterinary Microbiology and Pathology, Washington State University, Pullman, Washington, United States of America; 5 Institute of Infection and Global Health, Department of Epidemiology and Population Health, University of Liverpool, Cheshire, United Kingdom; 6 Centre for Infection Immunity, and Evolution, Institute for Immunology and Infection Research, School of Biological Sciences, University of Edinburgh, Edinburgh, United Kingdom; 7 College of Natural Sciences, Makerere University, Kampala, Uganda; 8 Department of Agriculture, Food and Nutrition Sciences, University of Alberta, Edmonton, Alberta, Canada; 9 Department of Animal Science, Iowa State University, Ames, Iowa, United States of America; National Cheng Kung University, TAIWAN

## Abstract

The genetic diversity of African pigs, whether domestic or wild has not been widely studied and there is very limited published information available. Available data suggests that African domestic pigs originate from different domestication centers as opposed to international commercial breeds. We evaluated two domestic pig populations in Western Kenya, in order to characterize the genetic diversity, breed composition and admixture of the pigs in an area known to be endemic for African swine fever (ASF). One of the reasons for characterizing these specific populations is the fact that a proportion of indigenous pigs have tested ASF virus (ASFv) positive but do not present with clinical symptoms of disease indicating some form of tolerance to infection. Pigs were genotyped using either the porcine SNP60 or SNP80 chip. Village pigs were sourced from Busia and Homabay counties in Kenya. Because bush pigs (*Potamochoerus larvatus*) and warthogs (*Phacochoerus spp*.) are known to be tolerant to ASFv infection (exhibiting no clinical symptoms despite infection), they were included in the study to assess whether domestic pigs have similar genomic signatures. Additionally, samples representing European wild boar and international commercial breeds were included as references, given their potential contribution to the genetic make-up of the target domestic populations. The data indicate that village pigs in Busia are a non-homogenous admixed population with significant introgression of genes from international commercial breeds. Pigs from Homabay by contrast, represent a homogenous population with a “local indigenous’ composition that is distinct from the international breeds, and clusters more closely with the European wild boar than African wild pigs. Interestingly, village pigs from Busia that tested negative by PCR for ASFv genotype IX, had significantly higher local ancestry (>54%) compared to those testing positive, which contained more commercial breed gene introgression. This may have implication for breed selection and utilization in ASF endemic areas. A genome wide scan detected several regions under preferential selection with signatures for pigs from Busia and Homabay being very distinct. Additionally, there was no similarity in specific genes under selection between the wild pigs and domestic pigs despite having some broad areas under similar selection signatures. These results provide a basis to explore possible genetic determinants underlying tolerance to infection by ASFv genotypes and suggests multiple pathways for genetically mediated ASFv tolerance given the diversity of selection signatures observed among the populations studied.

## Introduction

The genetic diversity of pigs in the East African region has not been fully characterized, and their breed composition has been subject of considerable speculation. The genetic characterization of these pigs in relation to disease susceptibility is important in understanding the genetic determinants of disease tolerance, which may impact the design of appropriate control strategies. Genetic improvement could make a significant contribution to food security not only in Africa but also other tropical environments

The limited studies available have shown that East African pigs have a complex ancestry, with haplotypes from Asian, Far-eastern and European pigs all present in certain populations [1]. The genetic characterization of these pigs in relation to phenotypic traits, including pathogen susceptibility and productivity in resource-limited systems is important. Generation of genetically optimized pigs could make a significant contribution to food security in Africa

African swine fever (ASF) is a viral hemorrhagic disease of pigs (*Sus scrofa*) caused by the African swine fever virus (ASFv) that typically results in 100% mortality in naive animals, such as international pig breeds. In East Africa, the virus is maintained in ancient sylvatic cycle [2] that includes warthogs (*Phacochoerus spp*.) and *Ornithodoros* ticks. However, more recently pig to pig transmission, without involvement of wild pigs or suids is thought to be the major method of diseases dissemination in much of Africa. The disease has been recognized to be endemic in Africa since it was first formally identified in Kenya in 1921.

Bush pigs (*Potamochoerus larvatus*) have been shown to be resistant to ASF under experimental infection [2]. There is anecdotal and unpublished experimental evidence that local pigs in Africa are less susceptible to infection with specific ASFv genotypes than improved international breeds. Apparently healthy pigs have tested positive for the virus or viral antibodies, without clinical symptoms of the disease [3,4]. However, the determinants of this tolerance are not known. It is hypothesized that these pigs may have genetic tolerance which is absent from pigs domesticated in other regions of the world. This study sought to characterize the genetic diversity and genomic structure of local Kenyan pigs and relate these to the perceived tolerance to ASFv by comparing their signatures to those of known tolerant suids.

## Results

### SNP characteristics and genetic diversity

The term “local African” will be used throughout this paper to refer to a genetic signature characteristic of Homabay and Busia pigs, and distinct from “wild African” (bush pig and warthog) and “commercial” pig signature. A total of 658 animals were evaluated using 47,784 SNP markers Table 1.

**Table 1:** Sample populations, source project and sample utility in the project.

Population	Sample number	Sample source	Analysis	Genotyping Assay
Busia	117	PAZ	ASFv presence Genetic characterization	Illumina PorcineSNP60
Busia	87	EpiASF	Genetic Characterization	Illumina PorcineSNP60
Homabay	34	EpiASF	Genetic Characterization	Illumina PorcineSNP80
Warthog	34	EpiASF	Genetic Characterization	Illumina PorcineSNP80
Bush pig	8	EpiASF (Murchison Falls National Park, Uganda)	Genetic Characterization	Illumina PorcineSNP60
Bush pig Uganda	6	Murchison Falls National Park, Uganda	Genetic Characterization	Illumina PorcineSNP80
North American Landrace	25	Commercial Company	Genetic Characterization	Illumina PorcineSNP60
Yorkshire	99	ISU	Genetic Characterization	Illumina PorcineSNP60
Duroc	134	ISU	Genetic Characterization	Illumina PorcineSNP60
Large White Cross	100	ISU	Genetic Characterization	Illumina PorcineSNP60
European Wild boar	14	Wageningen University	Genetic Characterization	Illumina PorcineSNP60

PAZ, People Animals and their Zoonoses project; EpiASF, Understanding the epidemiology of African Swine fever project; ISU, Iowa State University

Observed heterozygosity was highest in the Yorkshire breed and Busia pigs and lowest in the warthog and bush pig populations Table 2. Similarly, the number of polymorphic markers was lowest in warthogs with only 2205 of the 34122 amplified markers being polymorphic and highest in the Busia pig population with as high a percentage as 99% of the retained markers being polymorphic. The Homabay pig population had the lowest number of polymorphic markers of all domestic pigs analyzed.

**Table 2:** Observed heterozygosity (Mean ± SD) in various pig groups evaluated.

Population	Sample size	Markers tested	Markers polymorphic	FIS	IBS	Ho
Homabay	32	36719	29037	0.14	0.78 ± 0.03	0.23 ± 0.10
Busia	194	46307	45950	0.08	0.71 ± 0.04	0.33 ± 0.14
Bush pig	10	38614	29304	0.64	0.99 ± 0.00	0.09 ± 0.12
Bush pig 2^a^	4	43837	26049	0.09	0.81 ± 0.10	0.28 ± 0.04
Warthog	34	34122	2205	0.48	0.78 ± 0.05	0.01 ± 0.00
Wild Boar	14	46423	31004	0.29	0.99 ± 0.01	0.18 ± 0.06
Duroc	134	46424	37370	0.00	0.79 ± 0.02	0.26 ± 0.08
Landrace	25	45177	42074	0.04	0.73 ± 0.03	0.32 ± 0.06
Large White	100	46620	43408	-0.02	0.75 ± 0.02	0.33 ± 0.11
Yorkshire	99	46341	44664	-0.01	0.73 ± 0.02	0.34 ± 0.06

FIS–Population Fixation index; IBS–Proportion of loci identical by state; Ho–Observed heterozygosity

^a^ These 4 samples were thought to be bush pigs at sample collection but turned out to be introgressed domestic pigs after genotypic analysis.

The IBS score illustrates relatedness within populations, Table 2 and was highest within the bush pig and European wild boar populations with values above 98%, with the commercial pigs having values ranging between 73% and 79%. The Busia pigs had the lowest IBS values at 71%, signifying substantial diversity within that population at, while the Homabay pigs had the second highest IBS value at 78% Table 2. Within population fixation index (F_IS_) was highest in the wild pig populations (bush pig, warthog and wild boar populations with 0.64, 0.48 and 0.29, respectively) and lowest in the Large White and Yorkshire populations. The Homabay population had high F_IS_ values (0.14) compared to other domestic pigs, which ranged between 0.08 to -0.02

### Population structure

**Fig 1:**
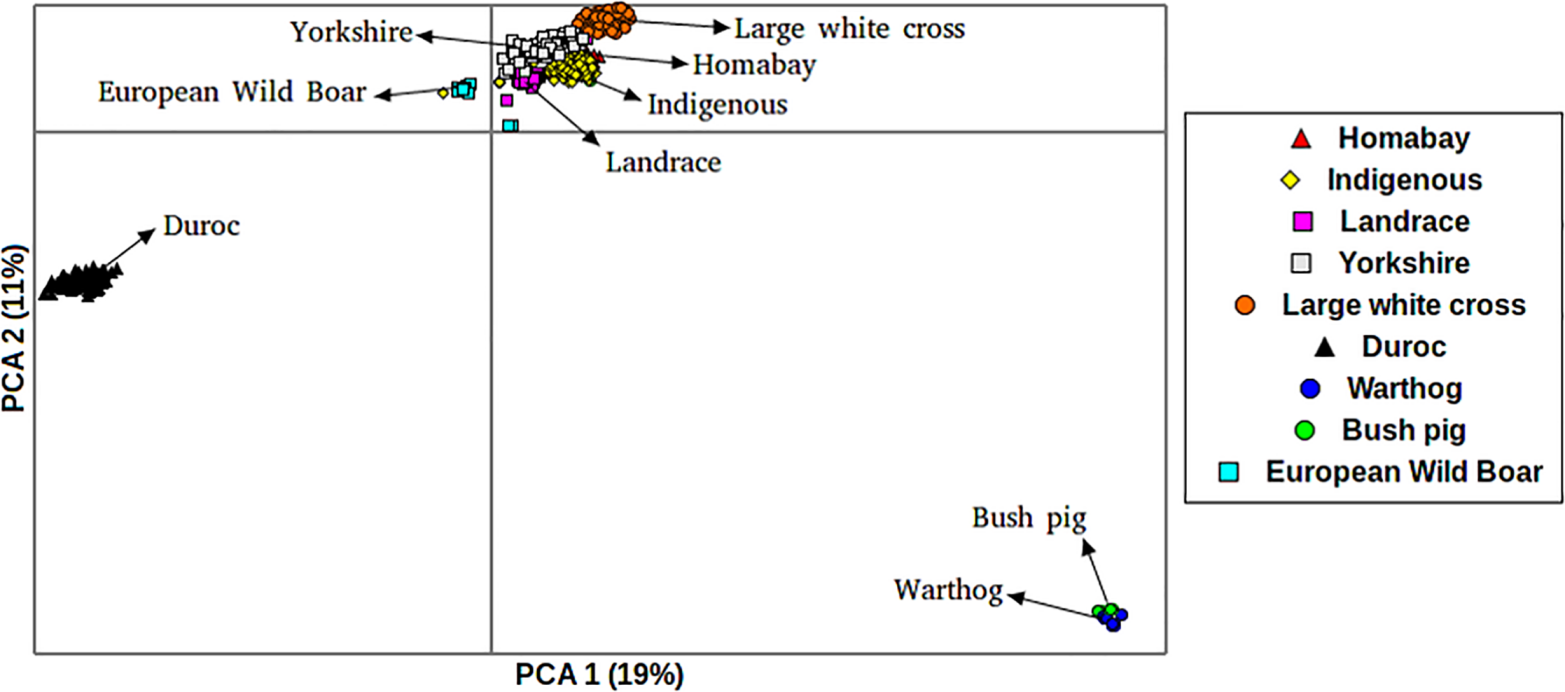
PCA plot for pigs indicating their distribution along the first two eigenvectors.

PCA was used to provide insight into the population structure of the local African pigs. Considering the first 3 PC’s a set of 6 clusters emerged. Homabay and Busia pigs were clustered separately. Bush pigs and warthogs were clustered together in a group distinct from the European wild boar. The last two clusters consisted of Duroc pigs (which formed a distinct group) and other commercial pigs. In Fig 1, the 1^st^ PC distinguishes the Duroc from the other suids while the 2^nd^ PC distinguishes wild African pigs from domesticated pig breeds.

**Fig 2:**
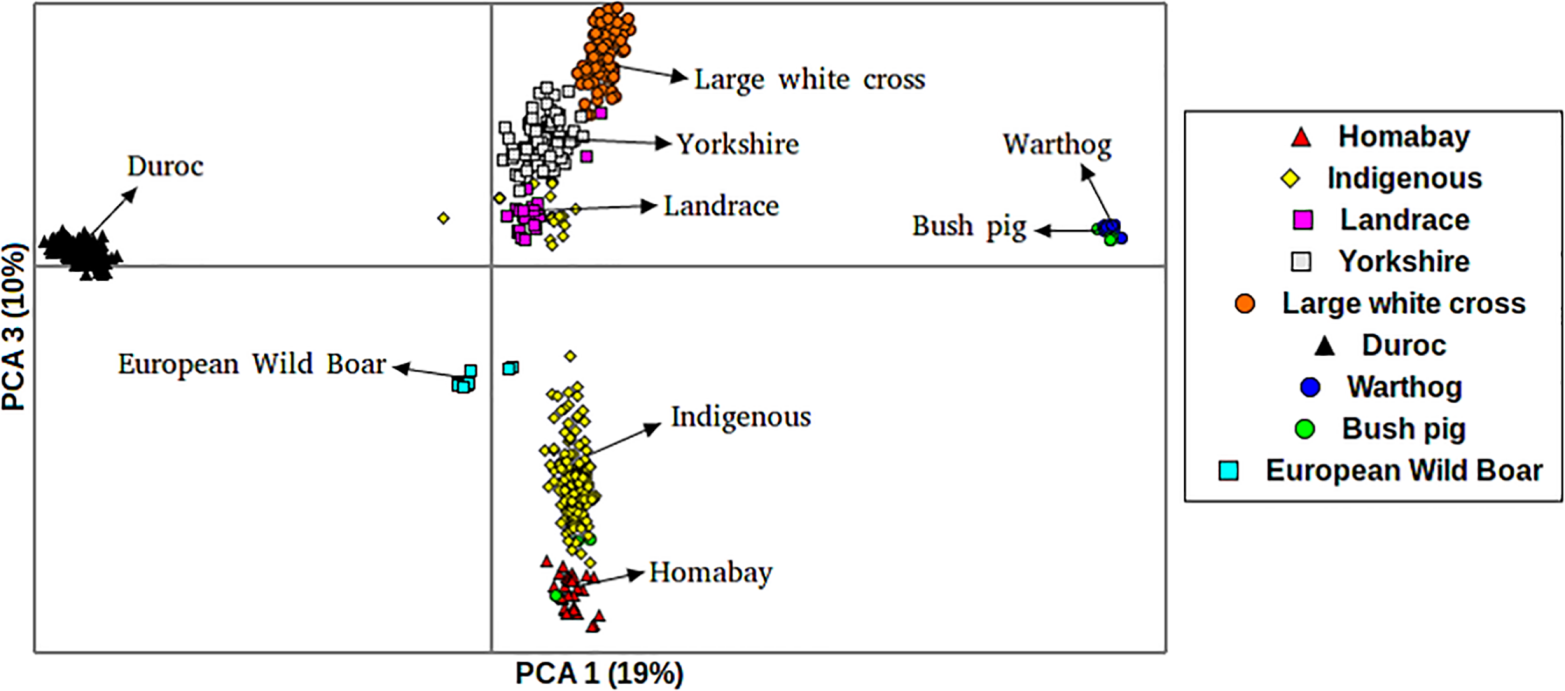
PCA plot for pigs indicating their distribution along the first and third eigenvectors.

The 3^rd^ PC distinguishes the African pig breeds from the international pig breeds (Fig 2). The European wild boar clustered more closely with the African pig breeds than with any other breed (Fig 2). The extent of genetic variation accounted for by the first 3 principle components was low at 40%, with PC 1, PC 2 and PC3 accounting for 19%, 11% and 10%, respectively, of the total variation.

### Admixture analysis

**Fig 3:**
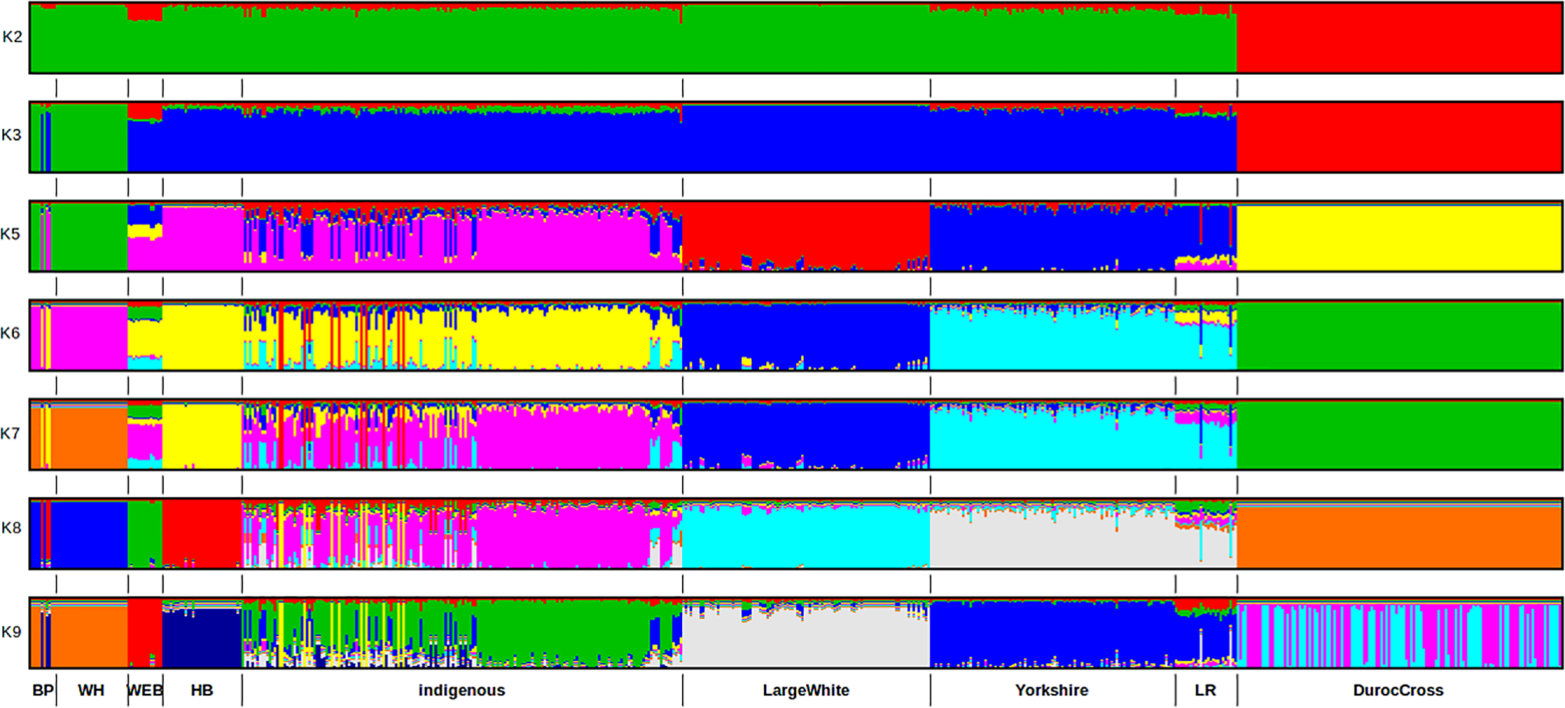
Admixture plot representing estimated membership coefficients for individual pigs for ancestral populations (K) ranging between 2 to 9.

**Fig 4:**
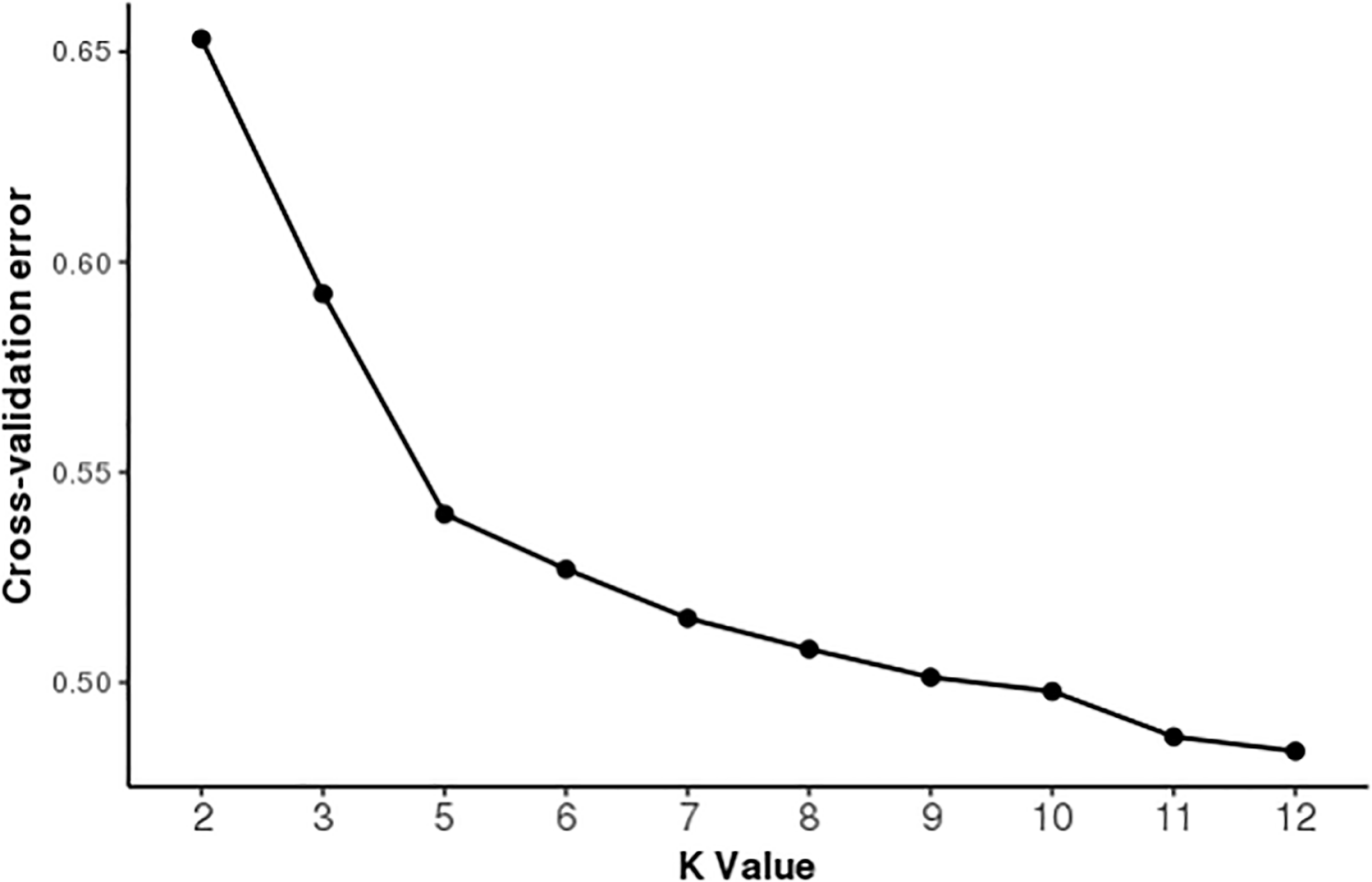
Cross validation plot indicating the model suitability as the number of putative populations (K) increases.

The ADMIXTURE runs from K = 2 to K = 9 are shown in Fig 3. The results indicate that the most likely partition was for K = 5 populations, based on visual inspection of the admixture plots. The change in prediction error against K (Fig 4) indicates minimal improvement in model fitness between K = 5 and K = 6, suggesting that K = 5 is the cluster number that best describes the study population.

At K = 2, the Duroc separates from the other suids, while at K = 3 the African wild pigs separate from the remaining pig populations. From the K = 3 analysis, it is clear that the African wild pig population is not a homogenous group, given that significant admixture (about 30%) is present within four bush pigs (three sampled from Gulu in Northern Uganda and one from Ruma National Park in Homabay) and four warthogs samples (from both the Maasai Mara National Park and the ILRI Kapiti plains Ranch).

At K = 4, local African pigs separate from the commercial breeds. The Busia pigs showed significant admixture, having introgression from commercial pigs. At K = 5, the Yorkshire and Large White breeds become distinct clusters within the commercial pig group. The results indicate that the Busia pig population consists of non-homogenous admixed pigs with an average commercial pig genetic composition of 10% (± 0.7% SE) and ranging from 0 to 28%. The Homabay domestic pigs displayed no trace of either wild or commercial pig introgression. Based on the cross-validation test, K = 5, seems to best describe the population Fig 4.

### Effect of genotype on ASF infection status

The population of pigs from Busia sampled as part of the PAZ project were tested for ASFv infection status. Differences in the mean composition, Table 3 and proportion Table 4 of animals that tested positive for ASFv were significantly associated with genotype. Animals testing negative had significantly (P = <0.0001) higher local African ancestry, (54% and above) compared to the ones testing positive for the virus. The proportion of wild African ancestry (bush pig or warthog) did not affect infection status since there was no significant difference (P = 0.5488) in the proportion of this ancestry in pigs positive or negative for ASFv. All infected pigs had ASFv genotype IX, the genotype responsible for most ASF outbreaks in Kenya [4,5].

**Table 3:** Minimum, maximum and average (least squares means with associated standard errors) ancestry composition for domestic pigs in Busia evaluated for ASFv (N = 117).

Infection status	ASFv Positive (N = 52)			ASFv Negative (N = 65)		
Ancestry	Min	Max	LS Mean ± SE	Min	Max	LS Mean ± SE
Local Busia	0.141	0.873	0.317 ± 0.025	0.548	0.887	0.763 ± 0.025
International commercial	0.127	0.844	0.675 ± 0.025	0.104	0.439	0.228 ± 0.024

ASFv–African swine fever virus; LS Mean–Least squares mean; Min–Minimum; Max–Maximum; N–Sample number; SE–Standard error

**Table 4:** The number of pigs that tested ASFv positive and negative given their local pig ancestry proportions.

Local pig ancestry proportion	ASFv Negative (N = 54)	ASFv Positive (N = 52)
< 25%	0	39
26–50%	0	4
51–75%	18	1
>75%	36	8

ASFv–African swine fever virus; LS Mean–Least squares mean; Min–Minimum; Max–Maximum; N–Sample number; SE–Standard error

### Selection signature analysis

**Fig 5:**
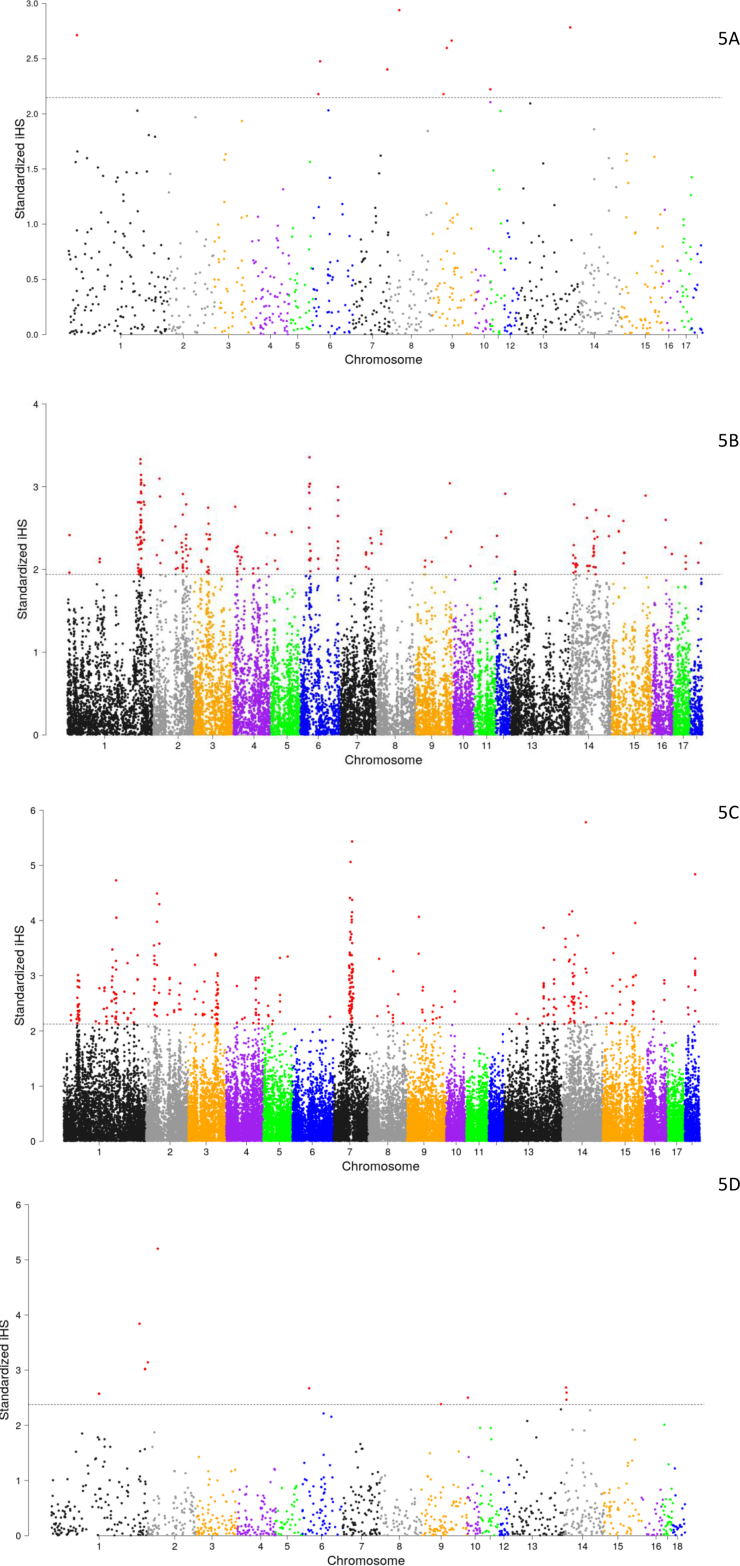
Plots of selective sweep patterns for various pig populations. The -log10(FDR-adjusted P) values are plotted against chromosome number. The dashed lines indicate the significance threshold for the top 1% SNPs based on with |iHS| value. Selective sweeps (iHS) for (5A) Bush pigs, (5B) Homabay, (5C) for Busia population and warthogs.

As shown in Fig 5A–5D, the patterns of selection based on integrated haplotype score (iHS) were quite distinct for the Busia and Homabay pig populations. Several large regions on SSC 1, 2, 3, 7, 9, 14, 15 and 18 seemed to be under differential selection in the Busia population, while regions on SSC 1, 6, 9, 12, 15 and 16 are potentially under selection in the Homabay population. None of the domestic pigs had selection signatures similar to the wild pigs.

Remarkably, based on these patterns, only 7 genes associated with SNPs ranked in the top 1% in terms of selection signal (iHS > 2.9) in Busia pigs were shared with the Homabay population’s top 1% and they were all from SSC 5 Table 5. However, a large number of genes was identified to be among the regions under selection across the Bush pig, Warthog, Busia and Homabay populations and the complete list is available in the S1 Appendix. The genes identified are involved in important pathways with presumed functions that spanned cellular signaling, inflammatory mediation, extracellular matrix functions, neural development and immune system activation as well as other processes such as translational, mitotic and endoplasmic reticulum-to-Golgi transport functions known to be important during infection events S1 Appendix. Additionally, the signal with the highest peak in the Busia population was on Chromosome 7 and was associated with the FOXG1 gene which encodes a protein known as forkhead box G1, a transcription factor. On the other hand, the highest peak for the Homabay population was associated with N4BP1, an interferon stimulated gene that has been shown to be important during viral infections [6].

**Table 5:** Genes located in regions under high differential selection and shared between Busia, Homabay and wild African pig populations.

Gene name	Chromosome	Description of gene function
AMHR2	5	This gene encodes the receptor for the anti-Mullerian hormone (AMH) which, in addition to testosterone, results in male sex differentiation.
MAP3K12	5	Mitogen-activated protein kinase kinase kinase 12. The gene encodes a member of the serine/threonine protein kinase family. This kinase contains a leucine-zipper domain and is predominately expressed in neuronal cells.
NPFF	5	Neuropeptide FF-amide peptide precursor; a putative receptor for RF amide-related peptides (RFRP).
SP1	5	specificity protein 1, a transcription factor
TARBP2	5	RISC-loading complex subunit: This gene encodes the receptor for the anti-Mullerian hormone (AMH) which, in addition to testosterone, results in male sex differentiation.
U6	5	U6 spliceosomal RNA
Uc 338	5	lncRNA ultra-conserved element 338 (uc.338) was first found to be upregulated in HCC and promote cell growth.

## Discussion

The terms ‘local’, ‘indigenous’ or ‘domestic’ are used loosely to depict pig breeds predominantly raised in East Africa and whose phenotypic characteristics are distinct from commercial breeds. It has been suggested that pigs in Eastern Africa were introduced through European contact [7] and as such are descendants of the Eurasian wild boar and should be related to international breeds, having descended from the same parental stock. The efficacy of using the Illumina PorcineSNP60 (and by extension Illumina PorcineSNP80) SNP chip on wild pigs has previously been successfully demonstrated, with several studies using this array for analysis [8]. Consequently, the SNP quality checks and inclusion criteria were less stringent in order to accommodate as many SNPs from warthogs as possible, given the low number of amplified markers in this wild pig species. In this study, we were unable to distinguish between bush pig and warthog using the SNP chips. Additionally, it was not possible to distinguish between the two species of warthogs (desert [*P*. *aethiopicus*] and savannah [*P*. *africanus*] warthogs) that were included in the study, probably due to the small number of informative marker loci that were successfully amplified Table 2.

The two SNP panels used in this study displayed significant ascertainment bias given that African pigs, which do not face intensive directional selection for productivity, are expected to have more genetic diversity than the international breeds, which is not the case in this study. The local African pigs from the Homabay region had genetic diversity measures that were lower than commercial pigs. On the other hand, because the Busia population comprised an admixture between alleles derived from African and commercial pig breeds, it had higher measures of genetic diversity, primarily driven by certain individuals with high proportions of commercial pig introgression.

The application of model based algorithms to determine population structure has dominated admixture analyses. However, we also used PCA, a classical nonparametric linear dimensionality reduction technique, in order to avoid making invalid assumptions about population composition or ancestry. The results from the PCA analysis are in concordance with those observed in the admixture analyses. The PCA defined six distinct clusters. It is important to note that the Homabay and Busia pigs clustered as two separate groups. In contrast, all commercial pig breeds (Landrace, Large White cross and Yorkshire) except the Duroc, clustered as one group.

Principle components accounted for a small proportion of the genetic variance in contrast with results from other species where the first two PCs account for a much higher percentage of the available variation. This may imply that a higher density marker panel is necessary to effectively describe African pig populations.

From the admixture analysis, the first hierarchical split is between all pigs and the Duroc. The clustering of African domestic pigs with the wild Eurasian boar is expected. What is interesting is that they cluster closer together compared to commercial pigs. Given our current understanding of the origin and history of pigs, it is widely held that the domestic pig originated from the Eurasian wild boar (*Sus scrofa*). The race native to North Africa is the *Sus scrofa algira*, whose habitat is thought to be along the Atlantic coast, as far as Rio des Oro in Western Sahara [9]. It is possible that descendants of the North African pig race spread downwards to the rest of Africa along the Nile basin. However, in Eastern Africa, the introduction of domestic pigs is believed to have been through contact with Europeans at colonization in the 18^th^ C. This means that local pigs in Kenya should share significant ancestral signatures with commercial pigs given the same wild progenitor. This is clearly demonstrated in the analysis with K = 2 and K = 3. However, several studies have shown that East African pigs have a complex ancestry, not only bearing European wild boar genetic components, but also those of Asian and far eastern wild boar [1]. This, together with the fact that commercial breeds have undergone intensive directional selection would explain why the local pig populations have distinct characteristics and cluster separately from the international breeds (Fig 2).

Recent evidence suggests that wild African suids are phylogenetically distinct from Eurasian *Sus* [10]. African domestic pigs should therefore be different from the African wild pigs. This is evident in the admixture analysis with K = 3 and K = 4. Additionally, the African wild boar is thought to be more closely related to the far Eastern wild boar than the Eurasian wild boar [11] and hence the two groups do not cluster together.

The Duroc pigs cluster separately from the other commercial pig breeds, as has been found previously [12]. The Duroc breed is thought to have been imported into America by Christopher Columbus from Spain or Portugal and then undergone further breed development in the late 1800s in the US. The clustering of the Duroc with African wild pigs’ hints at a possible European entry route through North Africa. The Duroc breed used in this analysis is thought to be a recent breed (Duroc-Jersey) developed in the USA [13], from pig populations of several ancestries (including Iberian and African pigs).

Results from the admixture analysis demonstrate that the bush pig samples analyzed do not come from one homogenous group. Bush pigs from Gulu in Uganda show some admixture with local domestic pigs (Fig 5). Additionally, four pigs that were included in the study as bush pigs, turned out to be domestic pigs. These pigs were admixed, containing between 5% to 9% wild pig introgression (Fig 5). We used mitochondrial cytochrome c oxidase I (COX 1) gene amplification to confirm the identity of the pigs. This classification error was due to hybrid pigs having phenotypic characteristics similar to the wild pigs. The wild pigs of Africa (warthog and bush pig) are generally thought not to hybridize with domestic pigs [9]. However, the presence of viable hybrids between bush pigs and domestic pigs has been recorded in South Africa, Congo and the Niger Delta, Nigeria [14]. This study adds credence to the possibility of viable hybrids between bush pig and domestic pigs. In order to study this possibility further, a higher density marker panel is required, given the very low levels of heterozygosity observed in the bush pig and warthog, Additionally, sequencing of mitochondrial loci would be valuable in determining the maternal lineage of the hybrid pigs.

We looked at runs of homozygosity based on iHS and selected genes associated with SNPs in the top 1% rank based on their | iHS| score. Significant regions under differential selection were observed Fig 5A, Fig 5B, Fig 5C and 5D. The Homabay pigs had a selection signature that was distinct from the Busia pigs and with very different sets of genes under selection S1 Appendix. This result is in concordance with the PCA plots that show these two populations as distinct groups. The difference in genetic structure was also observed through admixture analysis. The fact that genes identified under the regions seemingly under selection are involved in processes and pathways related to immune response and inflammation and should be considered as candidates for genetic determinants of ASF tolerance Table 5 and S1 Appendix. Additionally, it is significant to note that at present, bush pigs are a rare sight in Busia as compared to Homabay, where they are in abundance. The absence of such introgression in Homabay points to a clear demonstration of two populations of local pigs that have undergone divergent development to current status. Given the low number of amplified SNPs for the warthog, it may be necessary to undertake sequencing and/or SNP discovery in the wild pigs to allow for more accurate analysis of the selected regions

## Conclusions

The application of genome-wide SNP markers in characterizing the genetic diversity of domestic pigs revealed that local domestic pigs in Kenya are a non-homogeneous group. The Busia population consisted of admixed pigs of various breeds while the Homabay population represents pigs that are homogenous and whose composition is significantly different from that of the international commercial breeds. Detection of ASFv was correlated with pig genotype, with a higher proportion of pigs with low local African ancestry testing positive for the virus.

A comparison of the selection signatures between the local African and wild African pigs indicates absence of broad patterns of selection that are similar between the domestic and wild pigs. This may be due to the small number of markers that amplified for warthog and bush pig, limiting the ability to detect selection signatures with good accuracy. We also detected what appears to be evidence of introgression of bush pigs into domestic pigs. Given that wild African pigs are resistant to ASF, the presence of viable hybrids between domestic and bush pigs presents an opportunity to further characterize the genetic basis of ASF tolerance. However, a new marker panel would most likely be needed, given the sparse marker coverage and low genetic diversity observed in the wild pig population using the existing SNP panels.

## Materials and methods

### Site identification

The porcine samples used in this project were obtained from three sites by two separate projects. Samples from western Kenya were provided by the “People, Animals and their Zoonoses” (PAZ) project [15] and the ‘Understanding epidemiology of African swine fever’ (EpiASF) project funded by the former AusAID. Samples from Homabay, Nyanza province in Kenya were also provided from work funded by CISS-INIA Spain. All sites were within the Lake Victoria crescent ecosystem.

The warthog samples included in this study represent two different races; the desert and Savanna warthogs. The Savanna Warthogs (*P*. *phacochoerus*) were sampled in Mara National Park and Kapiti Ranch in Kenya. The desert warthogs (*P*. *aethiopicus*) were samples by KWS North of Garissa, North Eastern Kenya. Bush pig samples were obtained from Ruma National Park in Nyanza province and Gulu region in Uganda. Six additional bush pig samples were obtained from Makerere University (Uganda) having been sampled in Murchison Falls National Park in North Western Uganda.

### Data and sample collection

Table 1 summarizes information relating to the samples used in this study. Samples for the ASF epidemiology project (AusAID) were collected through a multi-stage stratified random sampling process. A cross sectional survey using a structured questionnaire was administered in selected pig keeping households in Busia county. Six hundred and twenty households from Busia were visited. The minimum sample size to be included in the study (320) was determined using the formula described by [16].

$$n=\frac{{\left(1.96\right)}^{2}P(1-p)}{L^{2}}$$

Where L is the required precision (+ or–error around estimate) at 5%, *P* the anticipated prevalence or proportion of attribute (set at 30%) and desired confidence level, *p* (at 95%). Data on general household information, production factors, health and disease management (tick control) was collected. A minimum of four pigs were targeted from each household so as to obtain a sample from a piglet, weanling, sow and boar. Only pigs that were older than 3 months were sampled to avoid mortality during blood collection by jugular puncture.

Sample collection for the ‘PAZ’ project is described in [4] who conducted a cross sectional survey of pigs at slaughter in Busia, and collected samples at designated slaughter houses. Blood samples were collected from the anterior vena cava into EDTA and plain 10ml BD Vacutainer® tubes with serum separation done at 3000rpm for 20 minutes at room temperature. Serum samples in 2 ml cryo-vials were cryopreserved at minus 20°C before laboratory analysis and at minus 80°C for long term storage. EDTA blood was used for DNA extraction. For the PAZ study, blood samples were collected from slaughter slabs.

### Viral DNA detection

Only porcine samples from Busia were analyzed for the presence of ASFv Table 1. Detection of ASF viral DNA was undertaken through a sensitive, gel—based PCR assay that is highly specific for detection of all the 23+ known ASFv genotypes [17]. This assay targets the highly conserved VP72 coding region of the ASFv genome. A positive sample was identified by the presence of a discrete band of 257 bp through UV visualization of PCR products on an ethidium bromide stained gel. The virus detection is described in greater detail by [4]

### Genotyping

A total of 216 random samples of local African pigs from both Busia and Homabay were selected from the pool of available samples for genotyping. A custom script was developed for this purpose in SAS 9.2 (SAS Institute, Cary NC). DNA samples from 34 warthogs and 14 bush pigs were also included in the analysis. Genotyping was undertaken at GeneSeek (Lincoln, Nebraska) using either the Illumina PorcineSNP60 or PorcineSNP80 bead chips (Illumina Inc., San Diego, CA, USA). The pig genome assembly *Sus scrofa* (SSC) build 10.2 was used to map the genomic positions of the SNPs. Additional genotypes representing pigs from American commercial lines were obtained from Iowa State University. These included the Yorkshire breed (100 samples), the Duroc breed (134 samples) and a commercial cross based on the Large White breed (100 samples). Genotypes for 25 North American Landrace pigs were provided by a commercial company, while genotypes of the European wild boar (14 samples) were contributed by researchers from Wageningen University in the Netherlands.

### Quality control

Quality control of SNP data was carried out using PLINK program [18] where individual samples were excluded if the number of missing genotypes were greater than 40%, while SNPs were removed if the missing genotype rate was greater than 20%. Only SNPs with a map position were included in the analysis. A minor allele frequency threshold of 0.05 was included to only retain the most informative markers while no Hardy Weinberg Equilibrium criteria (as introgression may result in loci not in Hardy Weinberg equilibrium) was applied. From the 64,000 (or 80,900 depending on the population) SNPs obtained after genotyping, a total of 46,177, common to both SNP arrays, and 618 animals met the inclusion criteria (across all sample populations) and were available for analysis.

### Admixture analysis

The program ADMIXTURE v1.2 [19] was used to evaluate population structure using all 46,177 SNPs, without excluding markers with high linkage disequilibrium (LD) values. The model employed in the ADMIXTURE program does not explicitly take LD into consideration but thinning of markers would have reduced the useful set of available markers for the wild African pigs to low marker numbers. Five independent replicates of the model were run for each cluster level (K = 2 to 12) in order to determine the cluster level with the best partitioning, in a five-fold cross-validation step. Breed composition classes were defined based on cluster membership probabilities obtained from the best supported K value in the admixture analysis. Each animal’s probability of cluster membership was used to group animals into 4 classes, where class 1 = <25%, class 2 = 25–50%, class 3 = 50%–75%, class 4 = >75% local African pig ancestry. The choice of the classes was informed by the target composition obtainable in a designed cross between a pure local African and a pure commercial line.

### Genetic relationships and population structure

The extent of genetic relationship was assessed quantitatively using IBS scores. Pairwise genetic distances within and between populations were calculated using the *ibis* function of GenABEL R Package [20].

### Effect of genotype on ASFv infection status

The association between infection status and breed composition was evaluated by comparing the proportion of animals testing positive among the breed composition classes for the 117 pigs from the Busia population which both genotyping and ASFv infection status data. The Cochran-Mantel-Haenszel (cmh) row means statistic was used to test the significance of the differences between the observed proportion in SAS 9.2 (SAS Institute, Cary NC).

### Selection signature analysis

Haplotypes were obtained by fastPHASE [21] with parameters K (number of clusters) = 15 and T (number of random starts) = 10. Analyses were run by chromosome for each individual. The derived haplotypes were then analyzed using the rehh R package [22] to compute the integrated haplotype score (iHS) [23] for each subpopulation. The top 1% of all SNPs were retained for gene analysis. Extraction of genes associated with the SNPs and gene function descriptions were obtained from the Ensembl gene annotation system [24].

## Supporting information

S1 Appendix List of genes identified in the top 0.25% iHS value for signatures of selection in Bush pig, Busia, Homabay, and Warthog populations. (XLSX)
